# The Multifaceted p21 (Cip1/Waf1/*CDKN1A*) in Cell Differentiation, Migration and Cancer Therapy

**DOI:** 10.3390/cancers11091220

**Published:** 2019-08-21

**Authors:** Nina-Naomi Kreis, Frank Louwen, Juping Yuan

**Affiliations:** Department of Gynecology and Obstetrics, University Hospital, J. W. Goethe-University, Theodor-Stern-Kai 7, D-60590 Frankfurt, Germany

**Keywords:** p21, *CDKN1A*, differentiation, migration, metastasis, anti-cancer therapy

## Abstract

Loss of cell cycle control is characteristic of tumorigenesis. The protein p21 is the founding member of cyclin-dependent kinase inhibitors and an important versatile cell cycle protein. p21 is transcriptionally controlled by p53 and p53-independent pathways. Its expression is increased in response to various intra- and extracellular stimuli to arrest the cell cycle ensuring genomic stability. Apart from its roles in cell cycle regulation including mitosis, p21 is involved in differentiation, cell migration, cytoskeletal dynamics, apoptosis, transcription, DNA repair, reprogramming of induced pluripotent stem cells, autophagy and the onset of senescence. p21 acts either as a tumor suppressor or as an oncogene depending largely on the cellular context, its subcellular localization and posttranslational modifications. In the present review, we briefly mention the general functions of p21 and summarize its roles in differentiation, migration and invasion in detail. Finally, regarding its dual role as tumor suppressor and oncogene, we highlight the potential, difficulties and risks of using p21 as a biomarker as well as a therapeutic target.

## 1. Introduction

p21, encoded by the gene *CDKN1A*, is the founding member of cyclin-dependent kinase (Cdk) inhibitors (CKI) [1], a pivotal cell cycle regulator ensuring genomic stability and often deregulated in human cancer [2,3,4]. Due to the lack of a defined tertiary structure, p21 is able to interact with a number of proteins involved in many key biological processes [5]. It is widely accepted that p21 acts through a “folding-on-binding mechanism” [6] with a prominent “binding promiscuity” [7]. As a critical effector of a variety of intra- and extracellular stress signals [8], p21 is exceedingly regulated by a myriad of transcriptional factors [9,10], distinct posttranscriptional regulators like microRNAs (miRNAs) and RNA binding proteins [11] as well as various posttranslational modifications. The short-lived p21 protein is degraded via different ubiquitin-dependent and -independent pathways [3,12]. Reversible protein phosphorylation by diverse kinases serves as an additional posttranslational mechanism controlling p21′s function, localization, binding partners, stability and degradation [3,13]. In particular, phosphorylation at distinct sites by various kinases can cause the cytoplasmic translocation of p21 greatly affecting its functionality and the therapeutic response of many chemotherapeutic interventions [2,3,4,14,15,16]. Importantly, p21 is not solely acting as a tumor suppressor but also as an oncogene depending on the cellular context leading to a negative or rather positive impact on tumor development and progression.

## 2. Functions of p21

### 2.1. p21 in General

The independent discovery of p21 by different groups leads to its numerous names [1,17,18,19,20,21], and reflects its multifaceted and heterogeneous cellular functionality. Rather than simply being a cell cycle inhibitor, senescence inducer and tumor suppressor, it is now appreciated as a much more complex and broader regulator [22]. It has additional key roles in apoptosis, differentiation, reprogramming of induced pluripotent stem cells, DNA repair, transcription and cell migration [2,3]. p21 is important for G2/M transition and mitotic progression, its loss prolongs mitosis resulting in mitotic defects and possibly in genomic instability [23,24]. p21 maintains chromosomal integrity in vivo [25]. As “linchpin” [26] or “molecular switch” [27] of cell fate, p21 is tightly involved in balancing and coordinating proliferation with all other cellular processes.

In general, p21 is an important regulator of cell cycle checkpoints ensuring proper cell division. p21 inhibits cell proliferation directly through binding to Cdks [1,18] and proliferating cell nuclear antigen (PCNA) [28], or indirectly at the transcriptional level [29,30,31]. Besides p53 [17], plenty of oncogenes, tumor suppressors, inflammatory cytokines and nutrients can initiate p21 transcription [9]. Interestingly, myelocytomatosis oncogene cellular homolog (c-Myc) has been shown to inhibit p21 gene expression [32] and, beyond that, it is able to interact with p21 disrupting its interaction with PCNA and causing a decrease in DNA synthesis inhibition [33]. The protective function of p21 is therefore disabled in tumor cells with high c-Myc levels [8]. In response to noxious environmental stimuli, p21 participates in the checkpoint control and initiates a temporary cell cycle arrest [34,35]. Using live single-cell measurements it has recently been shown that the amount of p21 and the time period of its increased expression contribute to the decision between cell cycle arrest and the repair of incomplete replicated DNA maintaining genomic stability [36].

Intriguingly, contradictory to its function as an inhibitor, low concentrations of p21 promote proliferation through the assembly and activation of cyclin D/Cdk4 or Cdk6 complexes in cancer cell lines [37]. This is supported by another study showing that primary mouse embryonic fibroblasts (MEFs) lacking p21 and its family member p27 failed to assemble detectable amounts of cyclin D/Cdk4 complexes [38]. Furthermore, hyper-phosphorylated p21 activates Cdk1 at G2/M transition [39]. Hence, the p21-describing term “cell cycle inhibitor” is a “confusing misnomer” [14]. The data highly suggest that p21 is not only an inhibitor but also an activator of the cell cycle depending on the cellular context and its expression levels.

Additionally, several studies revealed that p21 is involved in the major DNA repair pathways, which is attributed to its high PCNA binding capacity [28]. However, p21’s function in DNA repair is contradictory: p21 is reported to be inhibitory [40,41,42], unnecessary [43,44] or even required [45,46,47], mostly depending on the type of lesion/repair mechanism, the experimental setup, the cell system or/and the extent of DNA damage. A model was proposed in which the balance between p21 expression and DNA damage is crucial for the decision between DNA repair or rather apoptosis induction [48]. Low DNA damage tends to provoke a p21-dependent inhibition of apoptosis enabling DNA repair, whereas severe DNA damage causes the induction of apoptosis [48], probably through cleavage of p21 by caspase-3 [49,50].

The “antagonistic duality” of p21 [51] is particularly reflected in cell death mechanisms: p21 is regarded as a modulator of apoptosis [51,52] and is involved in macro-autophagy [53]. Cytoplasmic p21 leads to an inhibition of multiple caspases and apoptotic effectors including pro-caspase-3, caspase-8, caspase-10, apoptosis signal-regulating kinase 1 and stress-activated protein kinase [2,3]. Paradoxically, p21 can also promote apoptosis, as summarized in [52]. Notably, p21 activates autophagy to accelerate cell death in cancer cells [54,55], while it inhibits autophagy and induces apoptosis under death stimuli in normal cells [56]. Beyond that, p21 induces a senescent arrest in normal and tumor cells independent of p53 [57,58,59], identified through its overexpression in senescent human diploid fibroblasts [19].

In essence, p21 does not work solely as a tumor suppressor preserving genomic stability, permitting cell cycle arrest or senescence and enabling DNA repair. Meanwhile, there is increasing awareness of its oncogenic potential, often attributed to its cytoplasmic localization [3], promoting cell cycle progression, favoring migration [60,61] and inhibiting apoptosis [51], besides p21’s sustained (over)expression leads to bypassing/escaping from senescent cell arrest [62]. Its dual behavior in various processes often leads to opposing cellular responses (Table 1).

Of note, the individual roles of p21 should not be viewed in isolation. Instead, its multifaceted functions are interconnected and reciprocally influenced by each other.

### 2.2. p21 in Differentiation and Stem Cell Renewal

From its cDNA isolation, p21 is associated with differentiation [20]. Using subtractive hybridization, p21 was isolated based on its increased expression in human melanoma cells that were induced to differentiate, therefore, p21 was named melanoma differentiation-associated gene 6 (mda6) [20]. Given that cell proliferation and differentiation show a notable inverse relationship, their temporal coupling is crucial for growth and development, and is critical for tissue homeostasis [68]. Cell cycle arrest or rather withdrawal is a prerequisite and the first step towards terminal differentiation, and into quiescence or senescence, often accompanied by an increased p21 expression. Activation of p53, the major activator of p21 transcription [17], leads to cell cycle arrest via the suppression of cell cycle genes by the dimerization partner, RB-like, E2F and MuvB (DREAM) complex [29]. p21′s transcription is also activated during differentiation induced by p53-independent mechanisms through various agents including phorbol ester, okadaic acid, vitamin D3, tumor necrosis factor-α, myoblast determination protein, nerve growth factor together with aphidicolin, calcium, interleukin-6 or interferon-α [9].

Several lines of evidence indicate that p21 plays either a positive or negative role in differentiation depending on the cell type and the stage of differentiation [69]. Overexpression of p21 is able to induce cell differentiation in a variety of normal and tumor cells mediated by the induction of cell cycle exit [8]. Supporting its positive role in differentiation of normal cells, p21 promotes/establishes differentiation of murine oligodendrocytes [70], normal human peripheral blood monocytes [63], human and mouse endomitotic megakaryocytes [71], normal erythroid progenitors including reprogramming of murine erythroleukemia cells MEL [72] and murine C2C12 skeletal muscle cells [73,74,75]. Whereas muscular differentiation is impaired in p21-knockout mice [76], murine p21 expression correlates with cell cycle arrest and terminal differentiation of multiple cell lineages including skeletal muscle, cartilage, skin and nasal epithelium during mouse embryogenesis and adult tissue independent of p53 [77]. A rapid and transient increase of p21 was also observed in a temperature-sensitive fetal human intestinal (tsFHI) epithelial cell line, where it is involved in early stages of differentiation [78]. Furthermore, p21 has been shown to have an important but quite contradictory role in murine adipocyte differentiation: its knockdown in 3T3-L1 cells or its ablation in MEFs reduces adipocyte differentiation [79], suggestive of its pro-adipogenic role, while mice lacking p21 display adipocyte hyperplasia and obesity [80], implicative of its anti-adipogenic function. Moreover, murine stabilized nuclear p21 conveyed via phosphorylation at Thr-55 by murine protein serine-threonine kinase 38 (MPK38) inhibits adipocyte differentiation [81]. In line with this negative role, p21 inhibits differentiation of 32Dcl3 murine granulocytes [64]. It is strongly downregulated during differentiation of primary osteoblasts and osteoblastic cell lines derived from p21 null mice [82] and its expression decreases in terminally differentiated primary keratinocytes of mice [83]. Interestingly, p21’s expression elevates at the onset of keratinocyte differentiation [84] as reported for chondrogenic differentiation, in which p21 was increased at early stages of chondrogenesis and degraded in the later stages of murine ATDC5 cell differentiation [85].

Moreover, p21’s cytoplasmic localization plays a positive role for normal cell differentiation: the cytoplasmic residing of p21 was observed in mature human monocytes [63] and rat neurons [86], inhibiting apoptosis [63] or promoting neurite outgrowth through loss of stress fiber formation [86], respectively. Additionally, cytoplasmic translocation of p21 correlates with rat pancreatic myofibroblast to fibroblast conversion and protects against apoptosis [87]. During murine C2C12 myoblast differentiation, the appearance of the apoptosis-resistant phenotype is correlated with p21 induction [74] and its cytoplasmic localization in mature myofibrils through phosphorylation at Ser-153 [75]. These data strongly highlight the notion that p21 is tightly involved in normal cell differentiation, whether it confers a positive or negative role depends on a number of factors including its expression level, postmodification, cell type, differentiation stage and cell microenvironment (Table 2).

High attention has been paid to the link between p21 and differentiation/renewal of stem/progenitor cells. Interestingly, the reprogramming efficiency was highly increased upon depletion of p53 or p21 in MEFs [65,66]. The p53-p21-axis thus acts as a barrier and protector of induced pluripotent stem (iPS) cell generation since its silencing facilitates the reprogramming even in the presence of DNA damage [65,66,67]. In leukemic stem cells p21 prevents DNA-damage accumulation and exhaustion [88]. Increased p21 accelerates the differentiation of murine embryonic stem cells (ECSs) by repressing the pluripotency factor sex determining region Y-box 2 (*SOX2*) [89]. p21 is also indispensable for maintaining self-renewal of quiescent hematopoietic stem cells [90], keratinocyte stem cells [91] and neuronal stem cells [92] of mice. Therefore, inhibition of p21 results in the depletion of leukemic or hematopoietic stem cells, which is, depending on the cellular context, either positive or negative for the malignant progression [8]. Overall, basal p21 expression is required for stem cell maintenance protecting the cells from exhaustion, whereas high levels of p21 trigger differentiation and limit self-renewal of adult stem cells [93]. In addition, an up-regulation of cytoplasmic p21 accompanies differentiation of proliferating murine trophoblastic stem cells into giant cells, which are resistant to DNA damage induced apoptosis [94]. In contrast, studies have shown that human mesenchymal stem/progenitor cells (MSC) deficient in p21 have increased potential to differentiate into adipocytes, osteoblasts or chondrocytes [95,96,97], partially explained by a reduction of their replicative senescence [96,97]. Altogether, p21 is tightly linked to differentiation of various stem/progenitor cells (Table 2).

p21 is also involved in human cancer cell differentiation and development. In particular, epithelial–mesenchymal transition (EMT) and its reverse form mesenchymal–epithelial transition (MET) are crucial differential processes in cancer progression. While the activation of EMT permits cancer cells to acquire migratory, invasive and stem-like properties, MET is associated with the tumor-initiating ability required for metastatic colonization [98]. Markedly, p21 has been shown to attenuate EMT in cell lines and in mouse models: p21 inhibits EMT in colorectal cancer cells and normal human mammary epithelial cells (MCF10A) either in complex with zinc finger E-box binding homeobox 1 (ZEB1) through distinct miRNA clusters [99] or in response to transforming growth factor-*β* (TGF-*β*) [100]. p21 represses Ras- and c-Myc-dependent stem cell renewal and breast tumor EMT in mice [101]. Consistently, repression of p21 promotes EMT in response to long non-coding RNA plasmacytoma variant translocation 1 (PVT1) in distinct triple-negative breast cancer cell lines [102] as well as in cooperation with p53-upregulated modulator of apoptosis (PUMA) in MCF10A cells [103]. Silencing of p21 enhances the features of EMT in MEC cell lines, which are immortalized from the peripheral blood of a patient with B-chronic lymphocytic leukemia [101].

In further supporting p21’s role in cancer cell differentiation, the expression of p21 increases in various human malignant cell lines including lymphoma-derived cell line U937 [104,105,106], p53-null promyelocytic human leukemia (HL) cells HL-60 [107,108,109,110], acute myeloid leukemia (ML) cell lines ML-1, ML-2 and ML-3 [111], megakaryoblastic leukemia cell line CMK [112], epithelial colorectal adenocarcinoma cells Caco-2 [113], neuroblastoma cell line SH-SY5Y [114,115] and chondrosarcoma cells SW1353 [116], sometimes associated with its cytoplasmic localization [63,117]. In support of this, increased p21 was also observed in primary tumors from lung adenocarcinomas [118] and squamous cell carcinomas of the larynx [119]. Interestingly, p53-independent p21-overexpression is correlated with head and neck cancer differentiation [120]. In contrast, there is also evidence showing that p21 has an inhibitory role in cancer cell differentiation expanding its “antagonistic duality” [121] to this field. Stably overexpressed p21 displays decreased sensitivity to differentiation induction of human colon cancer cell line HT29 [122].

Loss of differentiation control can lead to a variety of diseases including cancer [68]. The entire evidence indicates that p21 plays a crucial role in differentiation of various normal or malignant cells and tissues but its impact relies on the cell type and the stage of differentiation. Of note, in the vast majority of studies, p21 has a positive role in differentiation; it initiates and/or is increased during cellular differentiation, partially by inhibition of apoptosis favoring cell survival. Unfortunately, it is not always clear whether the cells are terminally differentiated, in a quiescent or senescent state, since all these states are preceded by a G1 arrest mediated by p21 induction.

### 2.3. p21 in Migration and Invasion

Cell migration is a tightly integrated process orchestrating embryonic morphogenesis and contributing to tissue homeostasis [123]. Defects in cell motility are associated with pathological processes including metastasis responsible for over 90% of cancer associated deaths [124]. The role of p21 in cell motility is multifaceted depending on the cellular context. p21’s contribution to cell motility ascribes largely to its cytoplasmic localization, induced predominantly by phosphorylation of its distinct sites via various kinases [3,13]. Cytoplasmic p21 elicits an inhibition of the Ras homolog gene family, member A (RhoA)/Rho-associated, coiled-coil containing protein kinase (ROCK)/LIM domain kinase (LIMK)-pathway by binding to ROCK, increases cofilin expression and cytoskeleton remodeling due to a loss of actin stress fibers favoring cell motility in Ras-transformed MEFs [60,61]. The cytoplasmic accumulation of p21 requires firstly the oncogene Ras inducing its transcription [125] and secondly the phosphoinositide 3-kinase (PI3K)/protein kinase B (PKB, also named Akt)-pathway for its cytoplasmic retention [60]. Inhibition of PI3K, an upstream kinase of PKB/Akt mainly responsible for phosphorylation and cytoplasmic residing of p21 [3,126], causes the restoration of cofilin phosphorylation and a loss of cytoplasmic p21 [60]. Cytoplasmic p21 is also involved in neuronal differentiation regulating Rho-induced actin remodeling, which induces neurite outgrowth through suppression of actin stress fibers [86]. Interestingly, cytoplasmic p21 is targeted and degraded by Cullin-2 RING ubiquitin ligase with a leucine rich repeat 1 protein (CRL2^LRR1^) acting as a critical regulator of cell motility promoting a non-motile, stationary cell state by preventing p21 from inhibiting the RhoA/ROCK/LIMK pathway [127]. Knocking down Cullin-2 or LRR1 selectively elevates the protein level of p21 reducing stress fibers and cell-cell connections and raising F-actin on the cell periphery and cell motility in HeLa cells [127]. These morphological adjustments are suppressed by co-depletion of p21 [127]. Moreover, the accumulation of cytoplasmic p21 and p27 was linked to the oncogenic transformation of cells by activated Ras [60,128] or HER2/neu [129,130,131]. Importantly, cytoplasmic p21 has an oncogenic role in promoting mammary tumorigenesis and metastasis downregulating E-cadherin in vivo [132].

Oppositely, the complex of cytoplasmic p21 together with ectopic expressed cytosol-resided p53 (mutation in its nuclear localization sequence) suppresses cell invasion due to favoring apoptotic induction by targeting B-cell lymphoma 2 family proteins in non-small cell lung cancer cell line H1299 [133]. It has also been shown that the complex of p21 with wild type p53 induces Slug degradation further suppressing invasion [134]. In consistence with this observation, mutant p53 leads to Slug accumulation and increased cancer cell invasiveness [135].

p21 functioning as transcription factor/co-factor is essential for TGF-*β* mediated breast cancer cell migration and invasion, whereas its gene silencing blocked the tumor invasion in a mammary fat pad xenograft mouse model and various triple negative breast cancer cell lines, without alterations in cell growth and proliferation [136]. In this study high p21 expression was correlated with poor overall and distant metastasis free survival of breast cancer patients promoting migration/invasion at the transcriptional level [136]. Moreover, the complex of nuclear p21 and cyclin D1 is involved in actin remodeling of TGF-*β*-induced cell migration [137]. In accordance with this, another study with a breast cancer mouse model has shown that invasion is accompanied by an upregulation of p21 pointing to its role in a “reciprocal switching between proliferation and invasion” [138]. Together with p16, p21 promotes tumor growth in mice by enhancing the chemotaxis of monocytic myeloid-derived suppressor cells as observed with double-knockout mice [139]. Suppression of p21 compromises the migration and invasion capability of various trophoblastic and cancer cell lines mediated by, at least partially, a reduction of the extracellular signal-regulated kinase 3, matrix metalloproteinase 2 and tissue inhibitor of metalloproteinases 2 [140]. p21 knockout mice evince a dramatic suppression of metastasis, independent of tumor growth and rescuable by re-expression of p21 [138]. The long non-coding RNA small nucleolar RNA host gene 20 (SNHG20) represses p21 in non-small lung cell cancer cells A549 facilitating proliferation and migration [141]. Nuclear p21 inhibits but cytoplasmic p21 promotes migration and invasion in gastric cancer cell line AGS [142]. Recently, Galanos et al. utilized inducible p21-expressing p53-null osteosarcoma Saos2 cells and Li-Fraumeni patient-derived fibroblasts to study the impact of its sustained expression [62]. After 10 days induction, a population of cells bypassed senescence with increased genomic instability, chemoresistance and aggressiveness ascribed to enhanced invasiveness, partially explained by transcriptional upregulation of matrix metallopeptidases and “stemness-like features” [62]. The sustained p21-expression provokes radiation sensitive 52 (Rad52)-dependent error-prone double strand break repair promoting genomic instability [143].

The role of p21 in migration/invasion is associated with the environmental and cellular context, which is not unexpected, considering the reciprocal impact between versatile factors and p21.

## 3. Translating in Tumor Therapy

### 3.1. p21 and Its Deregulation in Cancer

p21 is often deregulated in human cancer. Depending on the cellular context, p21 promotes or inhibits tumorigenesis, extensively summarized by Abbas and Dutta [2]. p21 has been regarded as a tumor suppressor by regulating the cell cycle and maintaining genomic stability [2,3,4]. Though mutations in the p21 gene are regarded as extremely rare [4], the data from The Cancer Genome Atlas suggest that p21 is not infrequently mutated in bladder cancer (Philip Abbosh, Fox Chase Cancer Center) [144]. Some epigenetic alterations have also been reported [15]. Hypermethylation and silencing of p21 were observed in non-small cell lung [145] and prostate [146] cancer cells, high grade breast cancer [147] and acute lymphoblastic leukemia associated with poor prognosis [148]. Epigenetically silencing through long non-coding RNAs happens frequently [149] and was recently detected in cholangiocarcinoma [150]. Additionally, diverse single nucleotide polymorphisms of p21 are reported to influence the risk of developing cancers including esophageal [151], colorectal and estrogen-related cancer [152], associated with a higher risk of second primary malignancies in head and neck carcinoma [153].

p21 is often decreased in human cancer through loss of functional p53 or hyperactive oncogenes like c-Myc: p53 mutations have been discovered in more than 50% of human malignancies [154], whereas c-Myc, p21’s transcriptional repressor [32], is often overexpressed in human cancer [155]. Both drive p21 downregulation affecting its tumor suppressive activity and being linked to poor prognosis of patients with colorectal cancer, non-small-cell lung carcinoma, breast, gastric and ovarian cancers [2] as well as pancreatic cancer [156]. In consistent with these observations, p21 (over)expression has been correlated with a favorable prognosis in tonsillar carcinoma, gastric cancer, cervical adenocarcinoma, pancreatic cancer, as well as laryngeal and oral carcinoma [2].

In line with p21’s “antagonistic duality” [51], its overexpression leading to its oncogenic activity is found in a variety of human cancers including breast cancer, renal cell carcinoma, testicular cancer, hepatocellular carcinoma, multiple myeloma, gliomas, prostate cancer, cervical carcinoma, ovarian cancer, acute myeloid cancer, esophageal squamous cell carcinoma and soft tissue sarcomas [2,157]. Cytoplasmic p21 is observed in breast cancer [158,159] and hepatocellular carcinoma [160]. Recently, the role of p21 in gastric cancer samples was investigated and linked to its localization [142]. High cytoplasmic p21 level was positively associated with advanced tumor/lymph node/metastasis (TNM) stage, invasion depth, lymph node metastasis, distant metastasis and shorter overall survival of over 800 patients [142]. Cytoplasmic expression of p21 is acting as an oncogene with “anti-apoptotic gain-of-function” pointing to a role in tumorigenesis [161,162]. This residing appears frequently in human tumors, which was linked to aggressiveness as well as poor prognosis [2,4,14], and affected the response to chemo- and radiotherapy leading to drug resistance [15]. Taken together, the upregulation of p21 and its frequently cytoplasmic relocation correlate positively with poor prognosis, tumor grade, invasiveness and/or drug resistance. Despite the contradictory roles of p21 in the development of human cancer, increased cytoplasmic p21 is generally regarded as being tumor-promoting.

### 3.2. Lessons from Mouse Studies

Although p21 knockout mice were initially described to remain tumor-free until the age of seven months [163], a subsequent study showed cancer development at an average age of 16 month [164]. Evidence for classifying p21 as tumor suppressor came from additional deletion mouse studies. p21 knockout increased the rate of tumorigenesis in retinoblastoma protein [165] and adenomatous polyposis coli-haploinsufficient [166] and p18-deficient [167] transgenic mice, as detailed in [2]. Loss of p21 together with a mutation in p53 (p53R172P, preventing cells from apoptosis) accelerated the tumor onset [25]. In the context of mouse mammary tumor virus (MMTV)-Ras, p21 knockout also increased the tumor onset [168].

Conversely, p21 depletion in a c-Myc background decreased the overall tumor onset [168], and it reduced radiation-induced tumor development in wild type [164] and ataxia-telangiectasia mutated-deficient mice [169]. The absence of p21 resulted in a significant extension of the lifespan of p53-null and p53-haploinsufficient mice [170]. This effect can be attributed exclusively to a decrease in the incidence of spontaneous and radiation-induced thymic lymphomas [170] supportive of an oncogenic role of p21 in thymic lymphoma. Moreover, *Cdkn1a*;*Puma*;*Noxa* triple-knockout mice did not develop spontaneous tumors until the age of 500 days, suggesting the existence of additional pathways serving as mediators of p53-driven tumor suppression [171]. In sum, loss of p21 has differential effects on tumorigenesis based on the specific cellular context and the genetic background.

### 3.3. Considering p21 in Cancer Therapy

Given p21’s “antagonistic duality” [51] in various cellular processes (Table 1), it is obvious that p21 can have a dual role in tumor development and progression relying on the cancer type, the p53 status and the used chemotherapeutics. It can serve as a biomarker for specific therapies or prognosis, partially depending on its subcellular localization. In fact, the induction of p21 has been used as a drug response parameter [16].

Simply interfering with p21 as anti-cancer therapy bears risks and undesired side effects. First, increasing p21 can cause senescence, a supposed permanent growth arrest [172], which was believed to be only tumor suppressive terminating tumor regression, and is now regarded as a tumor promoter [173]. Senescent cells secrete numerous soluble factors promoting tissue repair, invasiveness of neighboring cells, chronic inflammation and tumor progression [174], and contribute to the escape of drug-induced apoptosis [175]. We examined the therapeutic potential of p21 in the context of Poloxin, a well-studied Polo-like kinase 1 (Plk1) inhibitor [176,177]. Plk1, a highly conserved serine/threonine kinase with critical roles during mitosis, is overexpressed in various tumor entities serving as a poor prognostic marker [178] and is thus considered as a promising target for molecular cancer therapy [179]. Cancer cells without p21 showed a stronger mitotic arrest accompanied by proliferation inhibition, more DNA damage and apoptosis induction upon Poloxin treatment relative to cancer cells with functional p21 and p53, which displayed a cytoplasmic re-localization and an anti-apoptotic feature [176]. Interestingly, long-term treatment (four days) of HCT116 p21+/+ cells with Poloxin led to senescence, whereas strong apoptosis induction was observed in cells lacking p21 [48]. Similar effects were detected in HCT116 cells treated with low doses of the anti-cancer drug camptothecin for four days [180]. Conversely, there are studies where overexpressed p21 enhanced the apoptotic response upon cisplatin treatment [181,182,183]. Remarkably, cellular senescence contributes to therapy resistance [184] and an aggressive tumor relapse by undergoing an epigenetic reprogramming of senescent cells into a stemness-like state [173,185]. In support of this observation, sustained expression of p21 exhibits oncogenic functions in a p53-null background leading to escaping senescence and chemoresistance [62]. Cells bypassing senescence display an increased genomic instability pointing again to p21’s “two-faced involvement” as genome guardian versus genomic instability mediator [186]. This duality is commonly attributed to the cellular or environmental context in which tumors develop. The mechanistic basis underlying such context-dependent phenomena remains to be defined in most cases, and its elucidation is essential for both understanding cell biology and the rational design of cancer therapy [187]. Thus, for therapeutic approaches, simply increasing p21 may not be beneficial and could provoke opposite undesirable/unintended outcomes.

Second, considering p21’s role in the cell cycle, stem cell differentiation and EMT of tumor cells, depleting p21 may result in either tumor suppressive or oncogenic effects depending on the cellular context. Cancer stem cells have been suggested to promote tumorigenesis as “seeds for metastasis” [188]. p21 is indispensable for maintaining self-renewal of leukemia stem cells [88], and it is able to inhibit oncogene-induced EMT and breast tumor stem cells in transgenic mice [101]. In a study with five patient-derived glioma stem cell-enriched cell lines, the authors have reported that p21 and p27 operate both as tumor suppressors, limiting cell proliferation, but also as oncogenes, conferring cell resistance to DNA damage and developing drug resistance [189]. Further investigations are mandatory to delineate if suppression of p21 serves as a therapeutic target, though its antisense therapy radiosensitizes human colon cancer cells [190] and glioma cells [191] to apoptosis.

Third, targeting cytoplasmic p21 is highly promising, as it contributes to tumor cell survival by inhibiting apoptosis, promoting cell migration and causing drug resistance [3,14,16,157]. Cytoplasmic expression of p21, which is common in human tumors, is linked to aggressiveness and poor prognosis [2,4]. The cytoplasmic residing of p21 is provoked by different kinases at distinct phosphorylation sites (Thr-57 [192], Ser-130 [192], Thr-145 [126], Ser-146 [193] and Ser-153 [75,194]), as summarized in [3]. Accordingly, increased cytoplasmic p21 due to Akt phosphorylation at Thr-145 favors tumor progression, drug resistance and poor prognosis [126,195,196,197]. Cytoplasmic re-localization of p21 by Akt-induced phosphorylation in HER2/neu overexpressing cells was correlated to apoptosis resistance [126]. Blocking the Akt pathway results in nuclear localization of p21 restoring its function as cell cycle inhibitor [126]. A recent study has shown that phosphorylation of p21 at Thr-145 and Ser-146 by Akt enhances cell survival contributing to taxol resistance in glioblastoma cell lines [195]. Cytoplasmic p21 is also associated with poor response to tamoxifen treatment in breast cancer cells MCF7 [198]. Further strong evidence comes from a recent investigation of the impact of cytoplasmic p21 on tumorigenesis in vivo [132]: MMTV/neu mice expressing cytoplasmic p21 (T145D, mimicking the Akt phosphorylation) in the mammary epithelium had an accelerated tumor onset as well as enhanced lung metastasis indicating an oncogenic role of p21 [132]. Moreover, cytoplasmic p21 induced by Akt-dependent phosphorylation enhances chemoresistance in response to doxorubicin in triple-negative breast cancer cells SUM159 [196]. Increased cytoplasmic p21 is related to cisplatin resistance in testicular and ovarian cancer [199,200] and to failure of paclitaxel treatment in human nasal squamous carcinoma RPMI-2650 [197]. Additionally, cytoplasmic p21 mediates 5-fluorouracil resistance in colorectal cancer cells [201]. A model for cancer aggressiveness are the human mantle cell lymphoma cell lines REC1, G519 and JVM2, where p21 increases its level along with disease progression and is localized to the cytoplasm [202]. Interestingly, cytoplasmic p21 is locally degraded by CRL2^LRR1^ and the *LRR1* gene is located at chromosome 14q21.3, which is frequently lost in metastatic cancer including breast cancer [127,203]. Accordingly, cytoplasmic localization of p21 is associated with anti-apoptotic properties and therapy resistance, which leads to a worse overall survival of patients suggesting p21’s prognostic importance.

Collectively, cytoplasmic localization of p21 could be a reliable biomarker and a promising intervention target. General targeting p21 will be associated with undesirable effects, regarding its multifaceted functions in stress-responsive senescence induction, apoptosis control, cell cycle regulation, genome integrity and stem cell maintenance.

### 3.4. Concepts of Targeting p21

**Figure 1:**
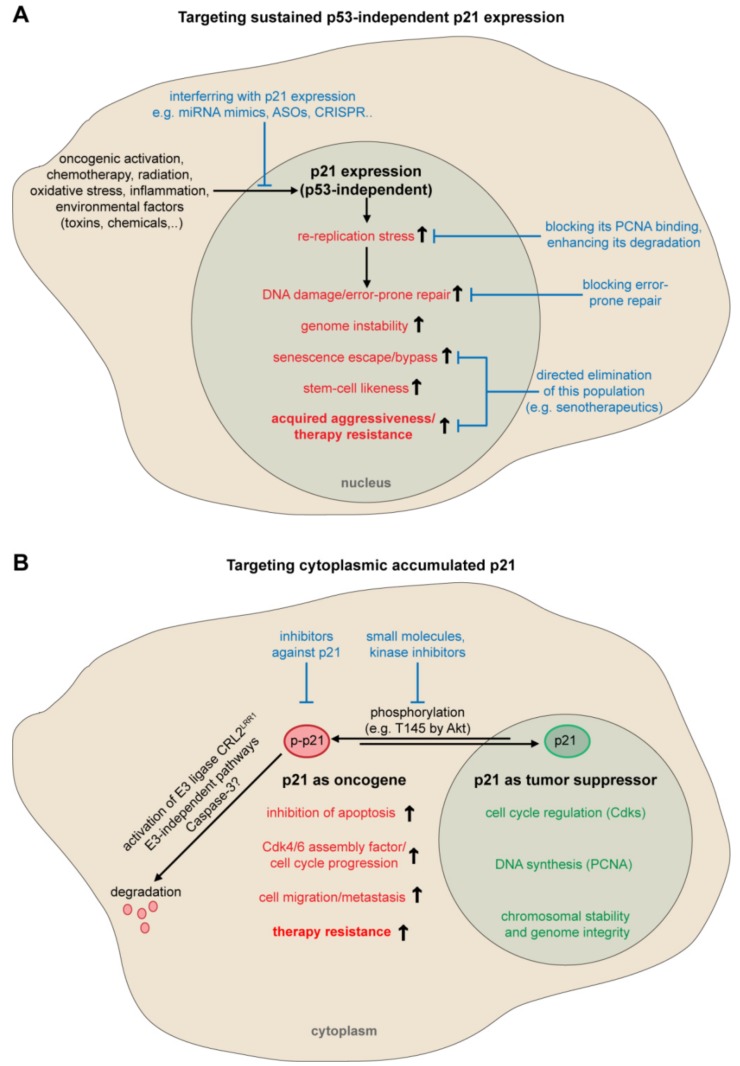
Schematic illustration delineating potential anti-cancer concepts by targeting p21. (**A**) Targeting oncogenic sustained p53-independent p21 nuclear expression. (**B**) Targeting accumulated p21 in the cytoplasm. Abbreviations: ASO, antisense oligonucleotides; Cdk, cyclin-dependent kinase; CRL2, Cullin-2 RING ubiquitin-ligase; CRISPR, clustered regularly interspaced short palindromic repeats; LRR1, leucine rich repeat 1; miRNA, microRNA; PCNA, proliferating cell nuclear antigen; p-p21, phospho-p21.

Given its differential expression and localization in human cancer, p21 could be targeted by three approaches [16]: first, increasing p21 expression, with histone deacetylase inhibitors [204] or through restoring p53 activity [205,206] as well as interfering with p21’s degradation using proteasome inhibitors [207], promotes its function as tumor suppressor and inhibits tumor growth. Second, down-regulation of p21 could be considered in malignant cells with highly expressed p21, especially with sustained p53-independent expression. Techniques of choice are antisense oligonucleotides (ASOs) [14], synthetic miRNA mimics [208,209] or prospectively a targeted therapy using the clustered regularly interspaced short palindromic repeats (CRISPR) technique [210]. ASOs targeting p21 has been already applied in breast cancer [211,212], colorectal cancer [190], glioma [191], myeloid leukemia [110] and renal cell carcinoma cell lines [213] showing growth arrest and/or apoptosis. Given their lacking efficacy and delivery options, transient expression and off-target effects, it is difficult to translate ASOs or siRNA based therapies into the clinic [157,214]. Novel iron chelators are also able to decrease p21 expression with anti-proliferative effects [215]. Few inhibitors targeting p21 are known including the Cdk inhibitor butyrolactone [216] and the small-molecule LLW10 [217], both induce p21’s proteasomal degradation. Due to the poor specificity targeting p21, these inhibitors are probably not the best choice for a clinical application. Alternatively, a possible interfering strategy would be to reduce its nuclear oncogenic properties by blocking its PCNA binding with small molecules like T2AA [218], by blocking error-prone DNA repair [219] or by the targeted elimination of therapy-resistant senescent cells with senotherapeutics including senolytics and senoptotics [220] (Figure 1A). Third, another promising alternative is to target cytoplasmic p21 or interfering with its nuclear-cytoplasmic shuttling (Figure 1B).

Currently, the best approach would be to target cytoplasmic p21, to inhibit its anti-apoptotic properties and to sensitize chemotherapy resistant cancer cells, as demonstrated in renal cell carcinoma [157] or breast cancer [14]. Interestingly, the small molecule UC2288 [221], which was synthesized based on the chemical model of sorafenib, the multi-kinase inhibitor [222], attenuates p21 at the level of transcription and posttranscription, and subsequently decreases cytoplasmic p21 [157]. Given that its cytoplasmic localization has so far been mainly attributed to its phosphorylation by Akt kinase [3], Akt inhibitors would be a good choice facilitating p21’s nuclear relocation and sensitizing cells to chemotherapeutics like cisplatin [199]. Identifying specific inhibitors to selectively block the nuclear-cytoplasmic-shuttling of p21 or to promote its cytoplasmic degradation would be also excellent desirable targeting strategies. However tissue- and cell-type specificity could complicate a general therapeutic strategy. Nevertheless, targeting p21’s sustained high expression (Figure 1A) and its cytoplasmic localization (Figure 1B) could be beneficial for cancer intervention. Yet, caution should be taken in terms of its localization, the p53 status and/or other potential genetic lesions affecting p21.

## 4. Conclusions

Being known over 25 years, p21 is still a challenging and fascinating protein. Rather than simply being a tumor suppressor, p21 also has oncogenic potential, demonstrated particularly by its sustained expression and its cytoplasmic localization, leading to chemoresistance and tumor heterogeneity, and reflecting its dual functionality depending on the cellular and environmental context. A great body of clinical, preclinical and cell-based studies provides insight into its molecular understanding, very often accompanied by inconsistency and divergence. While p21 conveys its multifaceted roles and impacts various fundamental cellular activities including cell differentiation and migration, its own regulation is dynamically affected by intra- and extracellular events. p21’s expression and localization influence the cellular response to external stimuli including chemotherapeutics. These agents, in turn, impact p21’s expression, localization, postmodification, stability and functions. Due to its functional “duality” [51] and its “intrinsically unstructured” [7] feature, it is still a long way to go to uncover the various faces of p21 in normal and malignant cells. Further investigations are required to entirely elucidate its roles under distinct cellular and environmental circumstances with different genetic backgrounds and to define its application as a prognostic marker and a therapeutic target in individual tumor entities. Despite this, p21 already shows a great potential by providing additional prognostic information for the selection of patients for an adequate anti-cancer therapy. Many questions are however still open. To finally define p21 as an trustworthy intervention target is “a work in progress” [144].

## Figures and Tables

**Table 1:** Dual behavior of p21 is highly dependent on its subcellular localization (nucleus or cytoplasm). Nuclear p21 acts mainly as a tumor suppressor and cytoplasmic p21 as oncogene (* representative references are shown).

Function of p21	Localization	Tumor Suppressor/Oncogene	References *
Apoptosis inhibition/activation	Both	Oncogene/Tumor suppressor	[51,52]
Autophagy induction/inhibition	Cytoplasm	?	[54,56]
Cell cycle arrest	Nucleus	Tumor suppressor	[1,18]
Cell cycle progression	Cytoplasm	Oncogene	[37,39]
Chromosomal stability	Nucleus	Tumor suppressor	[23,24,25]
Differentiation	Both	Both	[63,64]
DNA synthesis inhibition	Nucleus	Tumor suppressor	[28]
DNA repair inhibition/activation	Nucleus	Both	[40,47]
Maintaining stem cell renewal	Nucleus	Both	[65,66,67]
Migration, cytoskeletal dynamics	Cytoplasm	Oncogene	[60,61]
Re-replication	Nucleus	Oncogene	[62]
Senescence induction/escape	Nucleus	Both	[57,62]
Transcriptional co-factor	Nucleus	Tumor suppressor	[29,30,31]

**Table 2:** Overview of p21’s function in normal cell differentiation.

Cell Line/System	Association of Differentiation	References
Erythroid progenitors	p21 promotes differentiation of normal erythroid progenitors	[72]
Human MSCs	p21 deficiency causes increased differentiation capacity	[95,96,97]
Human PBMs	Increased cytoplasmic p21 during differentiation	[63]
MEFs (iPS)	p21 depletion induces reprogramming of differentiated cells	[65,66,67]
Megakaryocytes	High p53-independent p21 level in endomitotic megakaryocytes	[71]
Mice (in vivo)	p21 expression correlates with differentiation	[76,77]
Murine pre-adipocytes	p21 has a contradictory role in adipocyte differentiation	[79,80,81]
Murine ATDC5	p21-upregulation in early skeletal cartilage differentiation stages	[85]
Murine C2C12	Induction of (cytoplasmic) p21 correlates with an apoptosis-resistant phenotype of differentiating myoblasts	[73,74,75]
Murine 32Dcl3	p21 inhibits differentiation of granulocytes	[64]
Murine ESCs	Increased p21 in ESCs accelerates differentiation into endothelial cells, hepatocytes and neurons by repressing *SOX2*	[89]
Murine keratinocytes	Decreased p21 in terminally differentiated primary keratinocytes but increased p21 at the onset of differentiation	[83,84]
Murine oligodendrocytes	Increased p21 is required for establishment of differentiation	[70]
Murine osteoblasts	Strongly downregulated p21 during differentiation	[82]
Murine trophoblastic stem cells	Increase of cytoplasmic p21 during differentiation into apoptosis-resistant trophoblast giant cells	[94]
Rat neurons	Neurite outgrowth and branching of hippocampal neurons by cytoplasmic p21	[86]
Rat pancreatic myofibroblasts	Translocation of p21 from the nucleus to the cytoplasm correlates with pancreatic myofibroblast to fibroblast cell conversion	[84]
tsFHI	p21 is involved in early differentiation stages in human fetal intestinal epithelial cell line	[78]

Abbreviations: ESCs, embryonic stem cells; iPS, induced pluripotent stem cells; MEFs, mouse embryonic fibroblasts; MSCs, mesenchymal stem cells; PBMs, peripheral blood monocytes; SOX2, sex determining region Y-box 2; tsFHI, temperature-sensitive fetal human intestinal.
